# New Approaches to Targeting B Cells for Myasthenia Gravis Therapy

**DOI:** 10.3389/fimmu.2020.00240

**Published:** 2020-02-21

**Authors:** Ruksana Huda

**Affiliations:** Department of Microbiology and Immunology, University of Texas Medical Branch, Galveston, TX, United States

**Keywords:** myasthenia gravis, experimental autoimmune myasthenia gravis, B cell, AChR, MuSK, autoantibody

## Abstract

Current therapies for myasthenia gravis (MG) are limited, and many investigations have recently focused on target-specific therapies. B cell-targeting monoclonal antibody (mAb) therapies for MG are increasingly attractive due to their specificity and efficacy. The targeted B cell biomarkers are mainly the cluster of differentiation (CD) proteins that mediate maturation, differentiation, or survival of pathogenic B cells. Additional B cell-directed therapies include non-specific peptide inhibitors that preferentially target specific B cell subsets. The primary goals of such therapies are to intercept autoantibodies and prevent the generation of an inflammatory response that contributes to the pathogenesis of MG. Treatment of patients with MG using B cell-directed mAbs, antibody fragments, or selective inhibitors have exhibited moderate to high efficacy in early studies, and some of these therapies appear to be highly promising for further drug development. Numerous other biologics targeting various B cell surface molecules have been approved for the treatment of other conditions or are either in clinical trials or preclinical development stages. These approaches remain to be tested in patients with MG or animal models of the disease. This review article provides an overview of B cell-targeted treatments for MG, including those already available and those still in preclinical and clinical development. We also discuss the potential benefits as well as the shortcomings of these approaches to development of new therapies for MG and future directions in the field.

## Introduction

Myasthenia gravis (MG) is a chronic autoimmune neuromuscular disorder. Patients with MG who are seropositive for autoantibodies to the acetylcholine receptor (AChR), muscle-specific tyrosine kinase (MuSK), or low-density lipoprotein receptor-related protein 4 (LRP4) present with voluntary muscle weakness due to dysfunctional neuromuscular junctions and impaired neuromuscular transmission (1, 2). Traditional therapies for MG including thymectomy, intravenous immunoglobulin (IVIg), plasmapheresis, and corticosteroid therapy can induce remission, but do not cure the disease. Furthermore, 10–20% of patients remain refractory to immunosuppressive therapies (3). Hence, there is an immense need for new and effective treatments for MG, particularly refractory disease.

B cell-directed monoclonal antibody (mAb) therapies show great promise, and many are currently under development. These approaches primarily intend to eliminate or reduce the numbers of intact plasma B cells, or precursors of plasma cells, by blocking specific cell-surface biomarkers or cluster of differentiation (CD) antigens (4, 5). B cells have also been targeted indirectly, through inhibition of crucial molecules expressed by T-helper cells or other immune cells, or by inhibition of cytokines and chemokines that mediate affinity-maturation of B cells into plasma cells. Important membrane or signaling proteins that are exclusively or abundantly expressed in B cells or that contribute to their growth, survival, or overactivity in the context of autoantibody production and MG pathogenicity are typically regarded as B cell-specific therapeutic targets or biomarkers. To date, novel interventions designed to target such biomarkers have mostly comprised monoclonal antibody-based treatment approaches. Antibody fragment-based therapies have also been investigated, while peptide- or RNA-based therapies are less common, and many new potential therapies are rapidly emerging.

Both preclinical animal models and clinical studies in patients have provided abundant information on B cell-targeting therapies. A rodent model of MG (experimental autoimmune MG, EAMG), induced by immunization with Torpedo AChR, typically mimics clinical aspects of generalized MG (6, 7). Although in their early stages, the results from many preclinical studies are of considerable importance for future translational research and clinical application of new therapies. This review describes recent advances in targeted therapeutic approaches for MG, with a specific focus on B cell-targeted treatments for this disease.

## B Cells as a Target for MG Therapy

Both B cells and T cells are central players in the adaptive immune system. The signaling mechanisms that regulate B cell differentiation and activation processes are incompletely understood. Therefore, current B cell-directed therapies focus primarily on targeting intermediary (e.g., mature B cell subsets or plasmablasts) or terminally differentiated (e.g., plasma cells or memory B cells) B cell subsets with pathogenic implications.

### B Cell Biology and Subsets

**Figure 1:**
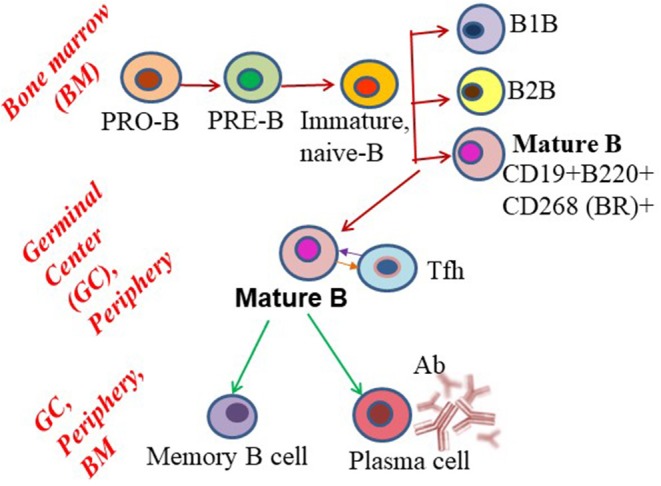
Schematic showing B cell subset differentiation. Pro-B cells progress through a series of developmental steps to generate mature B cells, which further differentiate into plasma and memory B cells.

B cell development begins in the bone marrow, the primary lymphoid organ, with the expression of B cell receptors (BCRs) in progenitor (Pro)-B cells (8, 9). Pro-B cells generate Pre-B cells, with complete light chain rearrangement and IgM expression. These Pre-B cells then leave the bone marrow to enter the peripheral circulation as transitional or mature B cell subsets. With the help of CD4^+^ T cells, mature B cells are activated to undergo somatic hypermutation and clonal selection, which generates follicular B cells in the secondary lymphoid organs, preferentially the spleen. These cells form germinal centers (GCs) and plasma cells that produce high-affinity class-switched antibodies, and memory and surrounding marginal zone B cells (Figure 1). Long-lived plasma cells stably maintain serum antibody levels, whereas memory B cells are responsible for recall responses upon antigen re-exposure (10). Most memory B cells and some long-lived plasma cells migrate to the bone marrow and replenish circulatory antibodies when needed. Although B cells reside predominantly in secondary lymphoid tissues (spleen and lymph nodes), during an infection or disease condition their numbers increase in peripheral blood and non-lymphoid tissues (11, 12). With the assistance of tissue-resident memory T cells, tissue-resident B cells may induce rapid plasmablast responses.

### Rationale for MG Therapies Targeting B Cells

MG is caused by autoantibody produced exclusively from autoantigen-specific plasma B cells (13). In approximately 80% of MG patients, the autoantigen is AChR, and the pathogenic autoantibodies are mainly IgG1 and IgG3 isotype anti-AChR autoantibodies (IgG2b in the EAMG mouse model). Anti-AChR antibodies cause generalized MG (affecting body muscles) and ocular MG (afflicting extraocular or eye muscles), which occur in ~85 and 50% of patients, respectively. Around 40% of patients seronegative for anti-AChR antibody, present with IgG4 isotype (IgG1 in mouse) antibodies against MuSK. An IgG2a autoantibody against the agrin receptor, LRP4, has also been detected in patients with MG seronegative for both anti-AChR and anti-MuSK antibodies (14). The mechanisms by which autoantibodies cause muscle pathology in MG have been well-described. Most anti-AChR autoantibodies recognize the main immunogenic region present in the α subunit of AChR, which is a four subunit protein. Binding of anti-AChR autoantibodies with AChR in the membrane reduces the availability of the functional receptor to ACh, either by blocking the receptor or leading to its internalization. Subsequent activation of complement cascades by the autoantibody leading to the formation of a “membrane attack complex”, also lyses myocytes (15, 16). Both MuSK and LRP autoantibodies can disperse postsynaptic AChR clusters and thereby cause AChR deficiency and muscle fatigue (17). Based on adequate evidence that autoantibodies cause MG development and progression through depletion of molecules critical for muscle function, as well as contributing to persistent inflammation, the majority of recent B cell-targeted therapies have focused on depleting B cells, thus reducing or removing the source of autoantigen-specific autoantibodies in patients with MG.

In addition to their main pathogenic role in autoantibody generation, B cells also serve as antigen-presenting cells to directly bind antigen on the BCR and present intracellularly processed antigenic peptides on their surface major histocompatibility complex (MHC) class II molecules (18). By upregulating costimulatory molecules, B cells then activate T cells to regulate their proinflammatory effector functions through secretion of a variety of cytokines, including tumor necrosis factor (TNF)-α, lymphotoxin, and granulocyte macrophage-colony-stimulating factor (19). In contrast, regulatory B cells (B-regs) secrete the anti-inflammatory cytokine interleukin 10 (IL10) contributing to B cell tolerance. Given their pathogenic roles associated with autoantibody and inflammatory mediator production, B cells are considered a preferred target for therapeutic intervention in MG.

### CD Biomarkers for B Cell-Directed Therapy Approaches

The CD antigens are immune cell biomarkers designated at Human Leukocyte Differentiation Antigens Workshops, which are held worldwide. B cells constitutively express a variety of CD antigens on their surfaces, which define distinct B cell subsets, in association with one or more specific biological functions such as survival, adhesion, activation, or inhibition. Due to their differential expression or activation in disease states, CD molecules serve as valuable cell surface signatures for B cell-targeted therapies in clinical trials.

**Figure 2:**
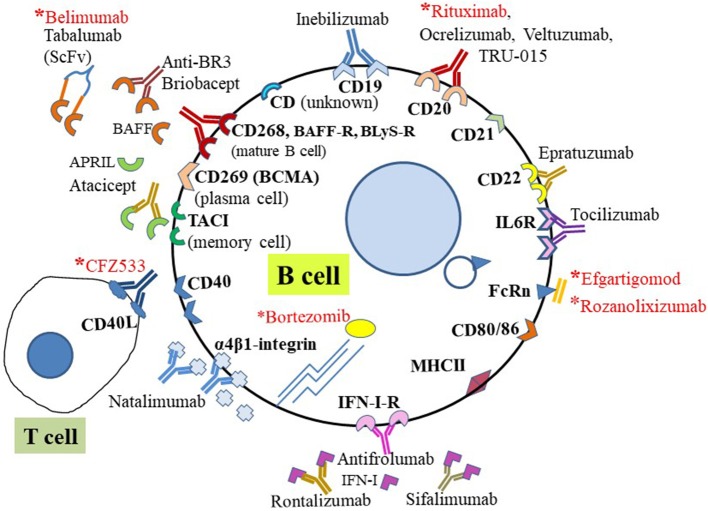
B cell-targeting therapies using CD surface biomarkers. Schematic representation of representative CD antigens expressed on the human B cell surface and targeted for B cell-specific therapy in autoimmune diseases. Those with asterisks (red) have been targeted for potential treatment of MG and are either approved for treatment or under investigation. For direct targeting, biologics (e.g., mAb or mAb fragments) directly bind cell surface CD molecules or receptors. Indirect treatments involve targeting soluble ligands of receptors.

To date, at least 58 CD molecules are established as expressed by B cells; these belong to the Ig superfamily (Ig-SF), tumor necrosis factor receptor superfamily (TNFR-SF), and cytokine receptor family. The Ig-SF includes five sub-families of CD antigens: Fc receptor-like (FCR-L), FCR, signaling lymphocytic activation molecule (SLAM), triggering receptors expressed on myeloid cells (TREM), and nectin. The following are among B cell-restricted CD antigens exclusively expressed (bolded) on B cells, and expressed either as a receptor or a ligand on B cells: CD10, **CD19**, **CD20**, **CD21**, **CD22**, CD23, CD24, CD27, CD37 to CD39, CD40, CD72 to CD78, **CD79a**, **CD79b**, CD80 to CD86, CD138, CD139, **CD179a**, **CD179b**, CD180, CD252, CD254, CD267 to CD269, CD275, CD307e, CD315 to CD317, CD307a to CD307d, and CD351 to CD363 (20, 21). Some therapeutically relevant B cell subtypes associated with exclusive expression of specific CD surface markers include plasma cells, which express CD269 and CD138 (Syndecan-1); mature B cells, expressing CD19, CD268, and CD79b; and memory B cells, with CD27. Many other B cell-specific CD antigens have been targeted (Figure 2), and those yet to be explored or targeted have potential for development as new diagnostic markers for MG therapy in the near future.

### Major Mechanisms Associated With B Cell-Targeted mAb Therapy

**Figure 3:**
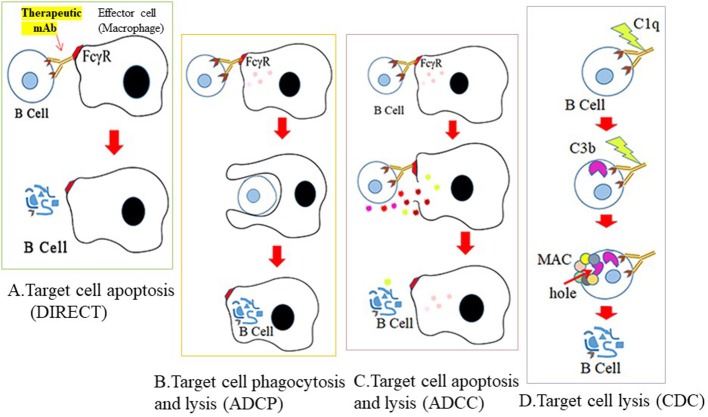
Mechanisms for therapeutic depletion of B cells. Therapeutic antibodies mediate their cytotoxic effects by four possible mechanisms. Cross-linking of mAb to B cell surface antigen **(A)** blocks ligand binding of essential receptors that mediate B cell survival (direct apoptosis), **(B)** triggers engagement of effector cells through recognition of its Fc sequence, and subsequent phagocytosis by effector cells (ADCP), **(C)** lysis by granzymes secreted from effector cells (ADCC), or **(D)** activates the complement cascade and lysis by C3 deposits and MAC formation on the cell surface.

The mechanisms by which CD antigen-specific mAbs mediate B cell depletion can be direct apoptosis of B cells, but often antibody-dependent cell-mediated cytotoxicity (ADCC) or antibody-dependent cellular phagocytosis (ADCP), while complement-dependent cytotoxicity (CDC) or cellular toxicity (CDCC) are also used (22) (Figures 3A–D). In ADCC, mAb binding to the B cell epitope is immediately followed by crosslinking of the fragment crystallizable (Fc) region of the mAb with the Fc receptor (FcγR) of effector cells (usually macrophages). The effector cells then polarize and release cytotoxic granules by perforin- or granzyme-mediated apoptotic pathways to destroy target B cells. During ADCP, once a mAb is bound to the target B cell, Fc-FcγR crosslinking activates effector cells, and the target cell is phagocytosed by the effector cell for intracellular destruction. In CDC/CDCC, binding of the Fc portion of B cell-bound mAb with C1q (a complement component) initiates activation of the complement cascade. C3b and C4b act as opsonins and subsequently form membrane-attack complexes (MACs) on target cells, to perforate the cell for lysis. Engineering the Fc arm of a mAb (e.g., glycoengineering and site mutagenesis) may further increase its effector function and serum stability (23).

## Clinical Approaches to B Cell-Targeted Therapy for MG

**Figure 4:**
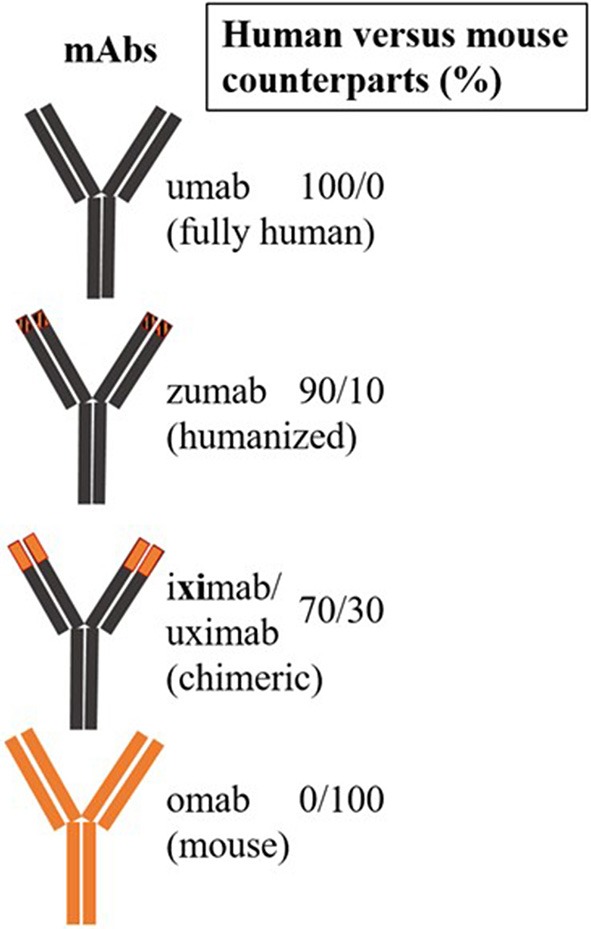
Diagram showing mAb characterization and naming for therapeutic use.

The first therapeutic mAb for targeted therapy in patients was a mouse anti-CD3 mAb, muromonab, used to prevent tissue rejection (24). Subsequently, mAbs have been engineered to incorporate both human and mouse sequences (humanized), followed by production of fully human recombinant mAbs. Chimeric (xi) antibodies consist of human amino acid sequences in the constant region, while humanized (zu) mAbs contain human sequences in the variable region. mAbs with partial chimeric and humanized sequences (xizu) or fully human (u) sequences have also been produced. The abbreviation “-ci (r)” is used to describe mAbs that target a circulatory system component, while li(m) denotes targeting of the immune system (e.g., -limumab) (25) (Figure 4). Many mAbs have been produced against different epitopes of the same CD molecule; however, their therapeutic potential has only been tested in one or a few specific diseases. Bi- or multi-specific mAbs that recognize two or multiple epitopes of the same antigen have also been developed. Single-chain variable fragment (scFv) molecules, consisting of fusions of variable regions of heavy and light chains connected by a short peptide linker, have also been used. Currently, the only additional non-mAb biologicals applied for targeted therapy are peptide inhibitors and recently generated antibody (Ab)-mimetics, which are ligand-specific, small synthetic proteins.

### Biologicals That Directly Target B Cells in MG

#### mAbs

##### CD20-targeting mAbs

Rituximab or RTX (also known as Rituxan, Rixathon, or Truxima [Genentech, San Francisco, CA, USA]) is a chimeric murine-human IgG1*k* mAb that targets CD20, a 33-kDa protein expressed on pro-B cells and all mature B cells, but not long-lived plasma or plasmablast cells. CD20 has an important role in the growth and differentiation of B cells into plasma cells, and rituximab can efficiently deplete CD20-positive B cells in MG patients; however, it is ineffective in reducing pathogenic AChR-Ab levels (26). Long-lived plasma cells are the major producers of autoAb and lack CD20, hence rituximab targets only short-lived plasma cells and CD20^+^, IL10-producing B-regs, or B10 cells, and reduction of autoAb is generally short term and insufficient, resulting in only transient clinical improvement (27). Thus, rituximab-treated AChR-MG and MuSK-MG patients often have disease relapse or recurrence after an initial phase of disease remission (28). Nevertheless, some studies have reported the efficacy of rituximab for treatment of MG, particularly MuSK-MG (29, 30). RTX was approved by USA FDA for treating refractory RA through intravenous infusion (31). It is also an off-label prescription for the treatment of refractory SLE, and has shown 51% complete remission, and 34% partial remission in SLE and Lupus nephritis (LN) patients (32).

##### CD40-targeting mAbs

Iscalimab or CFZ533 (Novartis Pharmaceuticals, Basel, Switzerland) is a fully human, Fc-silenced, IgG1 mAb that blocks the CD40 signaling pathway, thus preventing activation, but not causing depletion, of B cells and other CD40-positive cells. CD40 is expressed on B cells, T cells, and antigen-presenting cells, and its ligand, CD154, is primarily expressed on activated T cells (33). The CD40-CD154 interaction is important for isotype switching, GC formation, memory B cell generation, and Ab production (34). CFZ533 was evaluated as an add-on therapy for patients with generalized MG. A multi-center, randomized, double-blind, placebo-controlled clinical trial that measured quantitative MG muscle function scores has been completed, and the results are pending on Clinical Trials.gov.

##### FcRn-targeting mAbs

Beyond CDs, fragment crystallizable neonatal receptor (FcRn), an MHC class I-related receptor, was recently recognized as an important target in MG. This receptor is present on the cell surface and intracellular vesicles in many cells, including B cells, but not T cells. FcRn targeting has gained momentum in current therapies that aim to reduce pathogenic autoantibodies, as the receptor can inhibit cellular IgG degradation pathways that recycle IgG to maintain or elevate serum IgG levels (35). The receptor is also known to be involved in antigen presentation of peptides from the IgG immune complexes. Inhibition of FcRn with mAb or a mAb-fragment has shown promising results in reducing serum levels of pathogenic autoantibody in some autoimmune diseases, including MG; several trials are ongoing with the aim of establishing FcRn antagonists as a potent therapy for MG.

Efgartigimod (ARGX-113; Argenx, Breda, the Netherlands) is an FcRn antagonist investigational antibody fragment undergoing phase 3 ADAPT clinical trial for MG treatment. The therapeutic potential of ARGX-113 against immune thrombocytopenia and skin blistering diseases is also being evaluated. ARGX-113 is an Fc fragment of a CD70-specific recombinant Ab on a human IgG1 background (FR70-hIgG1) carrying mutations at residues specific for high-affinity binding to FcRn in B cells. The molecule blocks binding of circulating IgG to FcRn, thereby preventing IgG recycling and accelerating the removal of pathogenic IgG from the circulation and other cells. A single intravenous dose of ARGX-113 inhibited FcRn and caused a rapid and significant decrease in serum levels of IgG1, IgG2, and IgG3, but not IgD, IgE, IgM, or serum albumin, in patients with MG, relative to placebo (36, 37). In another phase 2 MG study involving 15 centers, three doses of ARGX-113 treatment in 1 month met both primary and secondary endpoints of tolerability, safety, and efficacy. This treatment rapidly decreased total IgG, anti-AChR Ab, and improved disease in 15% of patients (38).

Rozanolixizumab (UCB7665) is another lead candidate, humanized FcRn mAb. In a phase 2 trial completed in 2018, subcutaneous infusion of rozanolixizumab in patients with generalized MG significantly reduced anti-AChR autoantibody by at least 68% from baseline. Further development and recruitment of patients with MG for the phase 3 trial was initiated in 2019. Rozanolixizumab is also being evaluated for use in the treatment of immune thrombocytopenia (39).

Nipocalimab (M281), manufactured by Momenta Pharmaceuticals (Cambridge, MA, USA), is a fully human, recombinant anti-FcRn, glycosylated, IgG1 mAb. M281 received U.S. FDA-approved Fast Track designation for the treatment of warm autoimmune hemolytic anemia (phase 2/3) in the USA. It is also being evaluated in a phase 2 clinical trial (VIVACITY) which is a randomized, double-blinded, placebo-controlled, multi-dose trial including 60 patients with generalized MG; and the results are expected by mid-2020.

Ab-mimetics are ligand-specific small peptides of 3–20 kDa. They are analogous to the Fab arms of antibodies that lack Fc and are neither glycosylated nor immunogenic (40). ABY-039 (Alexion, New Haven, CT, USA) is a bivalent Ab-mimetic with a prolonged half-life form that exhibits high-affinity binding with FcRn. This therapeutic is currently under consideration for clinical trials.

### Biologicals That Indirectly Target B Cells in MG

#### mAbs

##### B cell-activating factor (BAFF)-targeting mAb

Belimumab (Human Genome Sciences Inc., Rockville, MD, USA; GlaxoSmithKline, Brentford, UK) is a human IgG1λ recombinant mAb that neutralizes the biologically active soluble form of BAFF, also known as B lymphocyte-stimulating factor, or BLyS, zTNF4, TNFSF13B, THANK, and TALL-1. Both membrane-bound and soluble forms of BAFF are produced by non-B cells; for example, monocytic and dendritic cells. BAFF binds with three different receptors: (1) Blys receptor 3 or BAFF receptor, which is predominantly expressed on mature B cells; (2) B cell maturation antigen (BCMA), which is exclusively found on plasma and memory plasma B cells; and (3) transmembrane activator and calcium modulator and cyclophilin ligand interactor (TACI), which is present on marginal zone and class-switched memory B cells. Based on preclinical experiments demonstrating that BAFF overexpression under autoimmune conditions induces autoreactive B cells that correlate with increased autoantibody levels, belimumab was developed to inhibit binding of BAFF to its receptor. BAFF also has a role in MG development and progression (41). Although belimumab treatment in patients with SLE had moderate efficacy in a multicenter phase 3 trials, treatment in an FDA-approved randomized MG study did not produce significant effects in patients with either AChR-MG or MuSK-MG (42).

#### Inhibitors

##### Proteasome-targeting inhibitors

Bortezomib (Velcade; Millennium Pharmaceuticals, Cambridge, MA, USA) is an FDA-approved proteasome inhibitor for treatment of cancer that has also exhibited clinical efficacy in MG (43). Further, bortezomib has can induce clinical improvement in SLE and has shown promising results in the treatment of MuSK-MG (43, 44). In the EAMG rat model, bortezomib reduced anti-AChR-antibody levels, prevented motor endplate damage, and induced clinical improvement (45). The inhibitor has also been shown to deplete plasma cells and specific autoantibody production in primary thymic cell cultures from patients with early-onset MG (46). Bortezomib allows cellular accumulation of misfolded or unfolded damaged protein, or unprocessed protein, that cannot be degraded or recycled or form a processed protein via proteasome pathway. Excessive build-up of such non-functional proteins leads to cell death (47). As plasma cells are actively engaged in producing autoantibodies in MG, significant accumulation of damaged and unprocessed proteins occurs, due to their rapid transcription and translation activities. Bortezomib can also hinder nuclear factor kappa-light-chain-enhancer of activated B cells (NFκB) activation through inhibition of IκB proteolysis, thereby suppressing transcription of NFκB-regulated genes (e.g., IL6, BAFF-R, etc.) (48). This drug has been assessed in a phase IIa trial on patients with therapy-refractory MG with significant disease activity, and the study result is currently awaited (49).

### B Cell-Targeting MG Therapies That Act via Blockade of Cytokines and Chemokines

Proinflammatory cytokines and chemokines (ILs) have major roles in MG pathogenesis. Inflammatory cytokines prime and activate dendritic cells, antigen-specific T-helper cells, and B cells, and induce pathogenic differentiation and development of plasma cells. Therefore, drugs designed to inhibit cytokine/chemokine activity also represent valid potential treatment strategies.

Tocilizumab (TCZ; RoActemra® or Actemra® Roche, Basel, Switzerland), also known as atlizumab, is a recombinant humanized mAb against the IL6 receptor (IL6-R). Of various cytokines that mediate Th1 and Th2 responses, IL6 plays a prominent damaging role in MG (50). Mice with an acquired or inborn deficiency of IL6 are resistant to MG, and anti-IL6 antibody reduces autoantibody levels and suppresses disease in a rat model of EAMG (50, 51). Although monocytes/macrophages are the main producers of IL6, it is also generated by numerous other cells, including muscle, epithelial, and B cells themselves. IL6 binds to IL6-R, CD126, or soluble IL6-R, and the ligand-receptor complex binds to CD136 (GP136), which dimerizes and subsequently activates intracellular kinases. Tocilizumab binds both cell-surface-bound and soluble IL6-R and prevents the proinflammatory effects of IL6. A published case report described two patients with MG refractory to rituximab treatment who showed clinical improvement after tocilizumab treatment, without any effect on autoantibody titer (52). Tocilizumab treatment has also demonstrated clinical effectiveness against RA, juvenile idiopathic arthritis, Castleman's disease, and Crohn's disease.

TNF is also produced at low levels by B cells. Conflicting results have been reported regarding the use of the TNF-α-inhibiting molecule, Enbrel® (etanercept, Benepali, Erelzi; Amgen, Thousand Oaks, CA, USA), and anti-TNF-α mAb treatment in MG and preclinical models. While etanercept (a fusion molecule containing the ligand-binding domain of human TNF receptor 2 and IgG1 Fc) was beneficial for patients with low plasma levels of IL6 and interferon (IFN)γ (53, 54), this TNF-α antagonist decoy receptor originally developed for treatment of RA, reportedly exacerbated MG in a patient with RA (55) and also reactivated tuberculosis in some patients (56, 57).

### B Cell-Targeting Potential Biologicals in Trial for Non-MG Autoimmune Diseases

This section describes some B cell-targeted potential therapeutics that have not yet been clinically tested for use in patients with MG. These drugs have shown promising results in early stage clinical studies for non-MG autoimmune diseases and therefore are potential pipeline drugs for future testing in MG.

#### Anti-CD19 mAb

Inebilizumab (MEDI-551) is a humanized high-affinity anti-CD19, IgG1κ mAb. Although plasma cells lack CD19 expression, this mAb induces effective ADCC to deplete almost all other B cells, including precursor-plasma cells. In phase 3, double-masked, randomized, placebo-controlled “N-Momentum” trial, inebilizumab demonstrated increased efficacy for the treatment of neuromyelitis optica spectrum disorder (NMOSD). In April 2019, the company Viela Bio received FDA Breakthrough Therapy Designation (BTD) approval for inebilizumab. BTD approval permits expedited development and fast regulatory review of drugs for life-threatening conditions, and those that achieve a minimum of one clinically significant endpoint. Inebilizumab has also received Orphan Drug Designation by both the FDA and the European Medicines Agency for treatment of NMO or NMOSD. This mAb has not yet been investigated for MG treatment.

#### Anti-CD20 mAb

Ocrelizumab (Ocrevus; Genentech), another CD20-binding humanized mAb, is FDA-approved for treatment of relapsing-remitting multiple sclerosis (RRMS) and primary progressive multiple sclerosis. Ublituximab (TG-1101; TG Therapeutics, New York, NY, USA) is a glycoengineered, B cell-depleting effective anti-CD20 mAb that has entered a phase 3 trial for treatment of MS. Veltuzumab (Immunomedics, Morris Plains, NJ, USA), a fully human anti-CD20 mAb, is currently under development for treatment of non-Hodgkin lymphoma and autoimmune diseases. TRU-015 (Trubion Pharmaceuticals Inc., Seattle, WA, USA: Pfizer Inc., New York, NY, USA), a fully human, anti-CD20 IgG fusion protein, is currently being evaluated for RA therapy. Anti-CD20 mAbs, such as: obinutuzumab (Gazyva; GlycArt Biotechnology AG, Schlieren, Switzerland; Roche), ofatumumab (Arzerra®), and tositumomab have been used in combination with chemo- or radiotherapy for the treatment of cancer.

#### Anti-IL6 mAb

Antibodies such as sarilumab, sirukumab, and siltuximab were developed to target IL6 for the treatment of RA or other diseases (58); however, these drugs have not yet been considered for use in the treatment of MG.

#### Anti-IFN mAb

Rontalizumab was developed by Genentech for treatment of SLE. It is a humanized IgG1 anti-IFNα mAb that neutralizes all IFNα subtypes and inhibits signaling through the type I IFN receptor (IFNAR). Primary and secondary endpoints were not met in the phase 2 trial. In a separate study, SLE patients with low IFN signature metrics who were treated with rontalizumab showed improvements in disease activity, reduced flares, and low steroid requirements (59). Sifalimumab, also a human anti-IFNα mAb, was developed by Medimmune (Gaithersburg, MD, USA). Recently, its trial was terminated by the company and replaced with a competing product, anifrolumab, for phase 3 trials. There are contradicting reports of the beneficial and adverse effects of IFN treatment in isolated case studies of patients with MG or EAMG experiment (60–62). As yet, IFN-I has not been targeted for MG clinical trial.

#### Anti-α4β1 Integrin mAb

Natalizumab (Biogen, Cambridge, MA, USA) is a recombinant humanized mAb that targets α_4_β_1_-integrin expressed by B cells. Natalizumab prevents binding of B cells to the endothelial adhesion molecule, VCAM, and consequently inhibits transmigration of B cells from the blood into tissues. Clinical trials revealed significant improvement and clinical efficacy in patients with RRMS treated with natalizumab.

#### BAFF and TACI Inhibitors

Anthera Pharmaceuticals (Hayward, CA, USA) developed another BAFF specific inhibitor, blisibimod, with high avidity against both soluble and membrane residing BAFF. In phase 2 clinical trial, blisibimod generated a response in SLE patients with disease severity (4). Further, in phase 3 trials of responder patients, the secondary endpoint was not met, although this treatment was associated with successful steroid-sparing and reductions in SLE autoantibodies and B cells. Atacicept (developed by ZymoGenetics, Seattle, WA, USA; handled by Merck Serono, Darmstadt, Germany) is a recombinant fusion protein that blocks the activation of TACI by APRIL and BAFF.

## Preclinical Studies of Potential B Cell Inhibition Using the EAMG Model

Many promising results of the use of B cell-targeted therapies for MG in mice models have been reported. These preclinical studies demonstrate the potential to target B cells for future translation in the clinic for MG therapy. The following section describes some preclinical studies that targeted B cells alone or in combination with other immune cells involved in MG pathogenesis.

### Proteasome Inhibitors

ONX-0914, a selective inhibitor of the immunoproteasome, has been reported to ameliorate EAMG severity by reducing the frequency of T follicular helper cells, antigen-presenting cells, and Th17 cells, as well as decreasing the affinity of B cell-generated autoantibodies (63).

### Chemokine Antagonists

Although MG is a B cell-mediated disease, B cell/T cell interaction plays a critical role in MG pathology. New therapies may also consider targeting factors involved in these specific interactions, particularly proteins belonging to the CC and CXC family of chemokine ligands and receptors, which are produced by peripheral blood mononuclear cells, lymph node cells, macrophages, and thymic GCs. Based on *in silico* analyses, potential therapeutic chemokine targets include: CXCR2, CXCR3, CXCL1, CXCL3, CCL, CCL19, and CCL20 (64–67).

### MicroRNA Inhibitors

MicroRNAs (miRNAs) are important immune regulators of numerous soluble inflammatory mediators and are potential targets for future intervention in MG. Differential levels of miRNAs in activated B cells (e.g., miR-146a) or serum have also been reported in an EAMG mouse model (68), and inhibition of these molecules reduced B cell activation and AChR-specific antibody levels. miR-150-5p and miR-21-5p are found at higher levels in patients seropositive for AChR, and their levels decrease following immunosuppressive therapy and thymectomy. Increased levels of the Let-7 family of miRNAs in MuSK-positive MG are also of great interest (69).

### Humanized scFv Against a DQB1 Allele Associated With Susceptibility to MG

In a preclinical study, a humanized scFv was developed from the mouse mAb, LG11, which targets MG-susceptible-specific human leukocyte antigen (HLA) alleles. The scFv was shown to block the proliferation of T cells cultured from peripheral blood lymphocytes from patients with MG carrying DQB1^*^0601, which is associated with susceptibility to MG (70).

### Recombinant AChR Fragment

Induction of tolerance by adoptive transfer of autologous regulatory T cells (T-regs) or mucosal delivery of AChR is effective for treatment of EAMG (71); however, the usefulness of these treatments has yet to be fully evaluated clinically. Consonni et al. recently reported that repeated intranasal administration of microgram quantities of a fusion protein that carries the immunodominant peptide from AChR, mCTA1-T146, suppressed both induction of EAMG, as well as ongoing disease in mice. Treated mice showed increased preservation of muscle AChR and low levels of anti-AChR serum antibodies. Tolerance was induced by increased T-reg cell activation and upregulated expression of *Tgf*β, *Il10, Il27*, and *Foxp3* mRNAs in the spleens and lymph nodes of mice (72). Similar induction of tolerance in EAMG has been described in response to nasal or oral delivery of recombinant AChR fragment, presumably by skewing Th1 to Th2/Th3 immune responses (73).

### BAFF Receptor-Specific mAb-siRNA Conjugates

In a different approach to targeting specific B cells, a fusion mAb-siRNA conjugate was constructed using a small <7-kDa protein (protamine), that covalently binds with B cell-targeting mAbs through hetero-bifunctional linkers and forms stable electrostatic bonds with B cell-specific siRNAs. The mAb in the conjugate binds to the B cell receptor and, upon internalization by receptor-mediated endocytosis, releases siRNA from the complex into the cell for degradation of B cell-specific pathogenic mRNAs. Treatment of EAMG mice with these conjugates can markedly reduce B cell frequencies (74). In an ongoing study, conjugates consisting of innate immune-resistant, modified siRNAs are being evaluated for therapeutic efficacy.

## Perspectives: Advantages, Limitations, and Future Challenges for B Cell-Targeting Therapies for Mg

There is an immediate but unmet need for effective MG therapy, particularly for patients with refractory disease. The high specificity, less off-target effects, and long-lasting, robust effects of B cell-specific mAb therapy make it attractive and especially useful for inhibiting proteins that do not have binding pockets available for an inhibitor. With the advent of humanized and fully human mAbs, mAb therapy is currently used as a first-line or standard treatment among targeted therapy approaches for many autoimmune diseases. The introduction of novel engineered mAbs is further evidence of the important progress occurring in the field.

Although mAbs can produce long-term cell-specific effects, stand-alone mAb therapy has some limitations, including inadequate understanding of its *in vivo* mechanism of action, adverse effects, and non-sensitivity to therapy (e.g., unaltered autoantibody levels or clinical pathology) despite significant depletion of target cells, which have been observed in many patients with MG following various B cell-directed mAb therapies. Currently, a major challenge for mAb therapy for autoimmune disease is inefficient reduction of pathogenic antibody, which is usually the primary goal of therapy. Tissue-trafficking of pathogenic plasma cells or precursor plasma cells to distant sites following targeted therapy is likely one cause of resistance to complete elimination of those cells, autoantibody reduction, and disease activity. Another highly probable cause is therapy-induced expression of molecules such as type I IFNs, which stimulate antigen presentation and affinity maturation of antibody-producing cells (74). High-dose mAbs may impart some therapeutic benefits to patients, but they also cause severe side effects, and patients are at risk of contracting infections and may generate innate-immune or interferonogenic effects (74). Hence, determination of the optimal dose for each therapeutic mAb is critical.

An approach using a combination of potent mAbs for targeted therapy may be effective. Targeting more than one subset of B cells, multiple targets, or specific B cells alongside B cell-interacting T cells and B cell-activating soluble mediators in the microenvironment, may result in synergistic reduction of autoantibody levels and therapeutic benefits. Treatment with mAb alone (or mAb-based therapy) is often inconvenient for both preclinical and clinical assessment due to their prolonged manufacturing times and high-cost relative to small molecule peptide inhibitors or therapeutic nucleic acids. However, most inhibitors have non-specific modes of action, risking induction of peptide-specific humoral responses or loss of activity over time. Nucleic acid therapy using modified RNA should be considered to prevent interferonogenic innate immune responses or immunogenicity of the therapeutic itself. Combining or conjugating more than one therapeutic molecule may also greatly enhance the therapeutic potential of B cell-specific therapy.

Changes in specific saccharide or sugar residues (fucosylation and galactosylation) can significantly modulate mAb effector function. Enzymatic removal of fucose and addition of galactose moieties to IgG1 mAbs can markedly increase their ADCC activity (75). The mechanism involves preferential binding of Fc to activating-FcγRIIIa (relative to inhibitory-FcγR) on effector cells, as determined by *in vitro* natural killer cell-based assays. Site-selective glycoengineering of both the Fc and Fab domains of a chimeric anti-epidermal growth factor receptor (EGFR) mAb has recently been shown to lead to increased ADCC activity (76). Future strategies for targeted B cell therapies may further exploit these technological advances by producing engineered mAbs that can potentially enhance B cell killing function and therapeutic efficacy.

Development of promising therapies largely depends on success in preclinical studies. The EAMG mouse model is valuable for therapeutic evaluation and redesign of promising B cell-targeted MG therapies. The classical EAMG model, generated by Torpedo-AChR immunizations, is preferable to systems using passive autoantibody transfer and thymic engraftment models (77–80) due to its chronic nature and mimicry of human MG. However, disadvantages of this model are the delay and lack of homogeneity in the incidence and clinical grades of immunized mice, despite comparable levels of high-affinity anti-AChR antibody. Hence, in individual studies researchers may choose to immunize an excess of animals to bolster statistical power. Another drawback of the EAMG model is the rapid progression of symptoms following disease onset, leading to mortality in some mice and reducing the number of experimental animals and durability of long-term assessment of therapeutic agents. The traditional tedious and inefficient method of immunogen purification from Torpedo tissue is also inconvenient and can be improved by utilizing new technologies and biomolecules such as UltraLink Biosupport (ThrmoFisher, CA, USA), a high-performance resin for the neurotoxin coupling reaction. Improving this animal model for more effective exploitation in preclinical therapy development will be essential for successful intervention in the clinic and effective MG therapy. Regarding therapeutic evaluation in clinical settings, recruitment of patients with similar disease profiles and their subsequent retention, as well as the fluctuating nature of the disease, are often challenging for proper evaluation of therapy. Other factors that potentially affect the successful outcome of clinical trials include study length, cost burden, and regulatory aspects.

It is important to be vigilant for occurrence of adverse effects of immunotherapy for cancer in patients with MG or underlying mild or latent MG (81–83). For example, treatment of patients with MG with ipilimumab (anti-CTLA4 mAb) for melanoma and lung cancer had fatal consequences (84). Further, combination therapy using mAb or inhibitors against checkpoint proteins (PD-1, PD-L1, and anti-CTLA-4) to treat cancers can induce or exacerbate MG, or even cause patient death (85, 86). It is likely that the robust immune activation and inflammatory response triggered by checkpoint protein-specific mAbs, although critical for cancer therapy, is detrimental to patients with MG, due to rapid development of myasthenic crisis; however, patients with MG and associated conditions other than cancer, such as thyroiditis due to anti-thyroid antibodies, lupus with anti-nuclear antibodies, and neuro-myelitis spectrum disorder with anti-aquaporin-4 antibodies (5), do not show adverse reactions following B cell-directed therapy.

## Conclusions

Developing strategies to find or develop an effective therapy for MG and many other debilitating and potentially life-threatening autoimmune diseases is an important research priority. Many potent candidate B cell-targeted therapies for MG are less effective or unsuccessful at the preclinical and clinical phases of development. These failures are primarily caused by weak immunosuppressive responses, non-specific immunogenicity, or safety concerns. Furthermore, targeted therapies currently licensed for use are effective but have drawbacks. Therefore, identifying and developing safe and effective means to improve the efficacy of these crucial therapies are urgently required. More preclinical studies of MG are needed to validate both new therapeutics and those that have already been proven effective for related neuromuscular autoimmune diseases. Additionally, identification of new and validated target(s), repurposing other targeted therapies, administration of combination therapies directed at multiple targets, and targeting antigen-specific B cells rather than a pan-B cell approach, may help to turn these targeted-therapy approaches into effective methods for ameliorating MG.

Overall, recent advances in targeted-therapy approaches have contributed significantly to our knowledge that has subsequently led to a multitude of new therapeutic modulations and emerging therapy approaches. For example, the development of highly potent, non-immunogenic, engineered mAbs and synthetic alternatives to mAbs, such as Ab-mimetics (e.g., monobodies and nanobodies), and even small RNA therapeutics, is encouraging and offers hope. These approaches may soon lead to the production of additional next-generation targeted therapeutics and long-awaited effective interventions against MG.

## Author Contributions

The author reviewed the literature, contributed solely to the writing, and approved the manuscript for publication.

### Conflict of Interest

The author declares that the research was conducted in the absence of any commercial or financial relationships that could be construed as a potential conflict of interest.
